# Severe Acute Respiratory Syndrome Coronavirus 2 (SARS-CoV-2) Membrane (M) and Spike (S) Proteins Antagonize Host Type I Interferon Response

**DOI:** 10.3389/fcimb.2021.766922

**Published:** 2021-12-07

**Authors:** Qi Zhang, Zhiqiang Chen, Chenxiao Huang, Jiuyuan Sun, Minfei Xue, Tingting Feng, Wen Pan, Kezhen Wang, Jianfeng Dai

**Affiliations:** ^1^ Institutes of Biology and Medical Sciences, Jiangsu Key Laboratory of Infection and Immunity, Soochow University, Suzhou, China; ^2^ Department of Nuclear Medicine, The First Affiliated Hospital of Soochow University, Suzhou, China

**Keywords:** SARS-CoV-2, COVID-19, antiviral immunity, evasion, IFN-I, signaling pathway

## Abstract

Coronavirus disease 2019 (COVID-19), caused by severe acute respiratory syndrome coronavirus 2 (SARS-CoV-2), has spread worldwide and has infected more than 250 million people. A typical feature of COVID-19 is the lack of type I interferon (IFN-I)-mediated antiviral immunity in patients. However, the detailed molecular mechanisms by which SARS-CoV-2 evades the IFN-I-mediated antiviral response remain elusive. Here, we performed a comprehensive screening and identified a set of SARS-CoV-2 proteins that antagonize the IFN-I response. Subsequently, we characterized the mechanisms of two viral proteins antagonize IFN-I production and downstream signaling. SARS-CoV-2 membrane protein binds to importin karyopherin subunit alpha-6 (KPNA6) to inhibit interferon regulatory factor 3(IRF3) nuclear translocation. Further, the spike protein interacts with signal transducer and activator of transcription 1 (STAT1) to block its association with Janus kinase 1 (JAK1). This study increases our understanding of SARS-CoV-2 pathogenesis and suggests novel therapeutic targets for the treatment of COVID-19.

## Introduction

Severe acute respiratory syndrome coronavirus 2 (SARS-CoV-2) is a newly emerged virus that has caused the global pandemic of coronavirus disease (COVID-19) (Zhou et al., 2020; Zhu et al., 2020). As of November 2021, there have been more than 250 million confirmed COVID-19 cases and 5 million deaths in 2 years (https://covid19.who.int/). Fever, dry cough, and fatigue are the primary manifestations in patients with COVID-19; however, a certain proportion of patients have no obvious clinical symptoms after infection (Wu and McGoogan, 2020). In the past 20 years, several emerging coronaviruses, including SARS-CoV, Middle East respiratory syndrome coronavirus (MERS), and SARS-CoV-2, have posed great threats to human health (Assiri et al., 2013; Huang et al., 2020). SARS-CoV-2 is a positive-strand RNA virus that belongs to the enveloped coronaviridae β-coronavirus. The viral genome encodes 16 nonstructural proteins (nsp1-16), four structural proteins (spike [S], envelope [E], membrane [M], nucleocapsid [N]), and seven accessory proteins (ORF3a, ORF3b, ORF6, ORF7a, ORF7b, ORF8, and ORF10). The nonstructural proteins include several critical enzymes that are required for SARS-CoV-2 replication, such as nsp3 (papain-like protease), nsp5 (chymotrypsin-like, 3C-like protease), nsp12 (RNA-dependent RNA polymerase [RdRp]), and nsp13 (Helicase). The structural proteins form the SARS-CoV-2 virion and the accessory proteins facilitate viral infection and pathogenesis.

In response to the invasion of pathogens, the host has a highly complex and elaborate immune system. The host antiviral immune response is divided into two stages: innate and acquired. The innate interferon signaling pathway is the first line of defense against viral infections; it is initiated by the recognition of pathogen-associated molecular patterns (PAMPs), such as single-stranded RNA (ssRNA), double-stranded RNA (dsRNA), or DNA, which triggers the production of type I interferons (IFN-α/β) by infected cells (Meylan et al., 2006; Acharya et al., 2020; Li et al., 2020a; Park and Iwasaki, 2020). Retinoic acid-inducible gene I (RIG-I) and melanoma differentiation-associated gene 5 (MDA-5) are the major cytosolic receptors for viral RNA. Upon activation, RIG-I or MDA-5 activates downstream IKKϵ and TBK1 by interacting with the mitochondrial antiviral signaling protein (MAVS), leading to the phosphorylation of interferon regulatory factor, IRF3. Activated IRF3 translocates to the nucleus and activates IFN production (Fitzgerald et al., 2003; Liu et al., 2015). At later stages, IFN receptor heterodimers engage with IFN-α/β and activate Janus kinase 1 (JAK1) and tyrosine kinase 2 (TYK2). Activated JAK1 and TYK2 phosphorylate STAT1 and STAT2, which are recruited to the interferon receptor (Levy and Darnell, 2002). Activated STAT1 and STAT2 then heterodimerize and combine with IRF9 to form the ISGF3 complex. ISGF3 activates promoters containing interferon stimulated response elements (ISREs) and induces the transcription of interferon stimulated genes (ISGs), thereby exerting antiviral effects (Schoggins et al., 2011; Schneider et al., 2014).

Virus invasion elicits a host immune response; however, the virus can also escape the host immune response in a variety of ways, such as inhibiting the production and secretion of IFN and blocking IFN signal transduction (Totura and Baric, 2012; Kindler et al., 2016; Channappanavar and Perlman, 2017). Several studies have reported that several SARS-CoV-2 proteins play diverse roles in antagonizing the IFN signaling pathway. For example, nsp1, nsp6, nsp13, nsp14, nsp15, N, 3CLpro, ORF6, ORF8, PLpro, and M could inhibit the IFN-I production (Li et al., 2020b; Xia et al., 2020; Yuen et al., 2020; Moustaqil et al., 2021); and nsp7, nsp13, ORF7a, ORF7b, N, and ORF6 inhibit IFN-I downstream signaling (Li et al., 2020b; Xia et al., 2020). Among them, ORF6 inhibits not only the activation of IRF3 and STAT1, but also the nuclear translocation of IRF3 and STAT1 in cooperation with nuclear transport factors, such as KPNA2 or Nup98 (Miorin et al., 2020; Xia et al., 2020). Similarly, N protein can inhibit both RIG-I-mediated IFN-I production and STAT1/2 mediated interferon signaling (Chen et al., 2020; Mu et al., 2020). PLpro inhibits IFN-I production by cleaving IRF3 while 3CLpro inhibits IFN-I production by clipping inflammatory regulators (Moustaqil et al., 2021). M protein may inhibit the production of IFN-I by inhibiting the formation of the RIG-I/MAVS/TRAF3/TBK1 complex (Zheng et al., 2020).

Although these viral proteins have been reported to antagonize host IFN signaling, the underlying mechanisms by which they do so remain elusive; whether other SARS-CoV-2 proteins participate in these processes or use different mechanisms remains unknown. Here, we screened SARS-CoV-2 proteins that could antagonize IFN-I production and IFN-I signaling. We found that the M protein had no obvious effect on the phosphorylation of IRF3, but inhibited its nuclear translocation. In addition, we found that the S protein suppressed phosphorylation and nuclear translocation by interrupting the interaction between JAK1 and STAT1.

## Materials and Methods

### Cells and Viruses

HEK293T, HeLa, and Vero cells were obtained from the American Type Culture Collection (ATCC) (Manassas, VA, USA) and cultured in Dulbecco’s modified Eagle’s medium (EallBio, China) supplemented with 10% fetal bovine serum (EallBio, China). Cells were cultured at 37°C in a humidified incubator (Thermo Fisher Scientific, Massachusetts, USA) containing 5% CO_2_.

Vesicular stomatitis virus-green fluorescent protein (VSV-GFP), expressing GFP as a nonstructural protein, was provided by Dr. Chunsheng Dong (Soochow University, Suzhou, China). This virus was propagated in Vero cells whereas Sendai virus (SeV) was propagated in chicken embryos. Cells were infected at a multiplicity of infection (MOI) of 1 unless otherwise stated.

### Antibodies and Reagents

The following antibodies were used: rabbit anti-DYKDDDDK tag (ABclonal, Cat # AE005), rabbit anti-HA (CST, Cat # 3724S), rabbit anti-IRF3 (ABclonal, Cat # D199862-0100), rabbit anti-p-IRF3 (CST, Cat # 4947S), rabbit anti-TBK1 (4A Biotech co.Ltd, Cat# 4ab032308cs), rabbit anti-p-TBK1 (Absin, Cat # abs140019), rabbit anti-KPNA6 (ImmunoWay, Cat # YN3049), rabbit anti-STAT2 (CST, Cat # 72604S), rabbit anti-p-STAT2 (CST, Cat # 88410S), rabbit anti-p-STAT1 (CST, Cat # 9167S), rabbit anti-p-TYK2 (Absin, Cat # abs131318), rabbit anti-TYK2 (Absin, Cat # abs131318a), rabbit anti-Myc (Proteintech, Cat # I6286-I-AP), anti-rabbit IgG HRP-linked antibody (CST, Cat # 7074), mouse anti-DYKDDDDK tag (Bioworld, Cat # AP0007), mouse anti-GAPDH (Proteintech, Cat # 60004-I-Ig), mouse anti-VSV-G (Abgent, Cat # AP1016a) and HRP goat anti-mouse IgG (BioLegend, Cat # 405306). Alexa Fluor 488 goat anti-mouse IgG (Thermo Fisher Scientific) and Alexa Fluor 647 goat anti-rabbit IgG secondary antibodies (Thermo Fisher Scientific) were employed, as well as protein A/G beads (Bimake) and anti-Flag magnetic beads (Bimake).

The following reagents were used: transfection reagent and PEI power (Aladdin, USA), Protease inhibitor (Sigma-Aldrich, USA), and recombinant human IFN-α (PeproTech, USA).

### Plasmids

SARS-CoV-2 ORFs were amplified from the prokaryotic expression plasmids (kindly provided by Dr. Shengce Tao from Shanghai Jiaotong University) and then subcloned into a eukaryotic expression vector with a Flag tag or Myc tag, individually. Human *KPNA2, KPNA3, KPNA4, KPNA6, RIG-IN, MAVS, TBK1, IRF3, IRF3/5D, STAT1*, and *JAK1* ORFs were amplified from the RNA of HEK293T cells and then individually subcloned into a eukaryotic expression vector with a Flag tag, HA tag, or Myc tag. The S1 and S2 subunits of the S protein were cloned into the eukaryotic vector, Myc-N1. The sequences of Oligo-primers used in this study were shown in **Table S1**.

### Real-Time Quantitative PCR

According to the manufacturer’s instructions, total RNA isolated with TRIzol Reagent (Takara) was reverse transcribed using a HiScript III 1st Strand cDNA Synthesis Kit with gDNA Wiper (Takara, Japan). Real-time quantitative PCR (RT-qPCR) assays were performed using a SYBR Green-based RT-qPCR Kit with SYBR Premix Ex TaqII (Vazyme, China) in a Roche LightCycler 96 system and amplified for 40 cycles (95°C for 10 s and 72°C for 30 s). The relative abundance of the indicated mRNA transcripts was normalized to that of *GAPDH*. The comparative CT (ΔΔCT) method was used to calculate fold changes in gene expression.

### Luciferase Reporter Assays

Fifty ng of viral protein expression plasmid, 50 ng IFN-β-Luc (or ISRE-Luc), and 5 ng pRL-TK were co-transfected into HEK293T cells seeded in 96-well plates. The pRL-TK Renilla luciferase reporter plasmid (Promega, USA) was co-transfected to normalize the transfection efficiency and used as an internal control. The cells were then infected with virus or stimulated with IFN-α (100 ng/µL). Cells were harvested after 36 h, and dual luciferase reporter (DLR) assays were performed using a luciferase assay kit (Vazyme, China) according to the manufacturer’s protocol. All reporter assays were performed in triplicate, and the results are shown as the mean ± standard deviation (SD) for each representative experiment.

### Coimmunoprecipitation (co-IP), Protein Mass Spectrometry, and Immunoblotting

For the Co-IP assay, HEK293T cells were harvested 36 h after transfection and lysed in lysis buffer (RIPA). Lysates were clarified for 10 min at 12,000 rpm at 4°C, incubated with anti-HA, anti-FLAG, or anti-Myc magnetic beads (Bimake, TX, USA), washed three times, and then incubated at 4°C overnight. Immunoprecipitates were eluted by boiling in 5× SDS loading buffer containing 0.2% (w/v) bromophenol blue, 20% (v/v) glycerol, 100 mM Tris-HCl (pH 6.8), 10% (w/v) SDS, and 1% (v/v) 2-mercaptoethanol.

For protein mass spectrometry, immunoprecipitates were digested with trypsin, and the mass spectrum of the peptide mixture was acquired using MALDI (EASY-nLC 1200, USA). The target peptides were identified by searching against the UniProt database of human proteins.

For Immunoblotting, samples are separated by sodium dodecyl sulfate polyacrylamide gel electrophoresis (SDS-PAGE), transferred to PVDF membranes, blocked with 5% (w/v) nonfat milk for 30 min, incubated with the indicated specific antibodies and corresponding secondary antibodies, then visualized with ECL western blotting detection reagent (Tanon, Beijing, China).

### Confocal Immunofluorescence Microscopy

HeLa cells were grown on 8-well slides and transfected with the indicated plasmids. After stimulation, cells were fixed with 4% paraformaldehyde, permeabilized with 0.1% TritonX-100, and blocked with phosphate-buffered saline (PBS) containing 5% fetal bovine serum. Slides were then incubated with the specific primary antibodies at 4°C overnight, rinsed, and incubated with the corresponding secondary antibodies. Nuclei were counterstained with DAPI (Abcam, Cambridge, UK). Images were acquired using a Nikon A1 confocal microscope (Nikon, Japan). The percentage of IRF3 or STAT1 in the nucleus were quantified by ImageJ software.

### Viral Titration

According to standard protocols, the titers of VSV-GFP in cell-free supernatants were determined with a median tissue culture infective dose (TCID_50_) assay using Vero cells. In brief, HEK293T cells were transfected with the indicated plasmids and then infected with VSV-GFP for 6 h. Culture supernatants containing viruses were serially diluted with DMEM and then placed on a monolayer of Vero cells in 96-well plates. The virus titer (TCID_50_/mL) of VSV-GFP was calculated using the Reed-Muench method.

### Flow Cytometry Analysis

At 6 h following infection with VSV-GFP, HEK293T cells were trypsinized and suspended in PBS with 0.5% BSA and 2 mM EDTA buffer. Thereafter, the cells were gated for GFP signals based on the background signal from the non-infected cells. Fluorescence intensity was determined using a Beckman Coulter Gallios flow cytometer (BD FACSCanto™ II Flow Cytometer 339473, USA) with at least 10,000 cells per sample. Data were analyzed using FlowJo™ software (BD Life Sciences, USA).

### Statistical Analysis

All data presented are representative of three or more independent experiments and are presented as mean ± SD. For statistical analysis, two-tailed unpaired Student’s *t-*tests were performed using GraphPad Prism 8.0 and Microsoft Excel. P values are shown in each figure or figure legend. In all cases, P < 0.05 was considered to be statistically significant.

## Results

### SARS-CoV-2 Proteins Antagonize the Host Innate Antiviral Response

**Figure 1:**
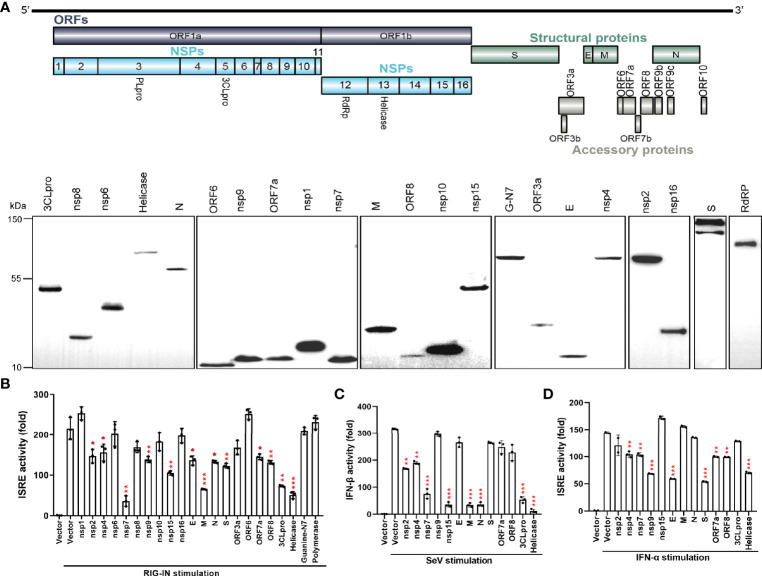
The SARS-CoV-2 proteins antagonize the host innate immune antiviral response. **(A)** Genome structure of SARS-CoV-2 (Strain Wuhan-Hu-1, GenBank MN908947) and the expression of SARS-CoV-2 proteins. C-terminally Flag-tagged viral proteins were expressed in HEK293T cells and analyzed by western blotting using anti-Flag antibody. GAPDH was used as a loading control. **(B)** Effect of viral proteins on ISRE promoter activation. HEK293T cells were co-transfected with the ISRE promoter-driven Firefly luciferase reporter plasmid, pISRE-luc, the Renilla luciferase control plasmid, pRL-TK, viral protein expressing plasmids, and the stimulator plasmid, RIG-IN. Empty vectors were used as controls. Cells were assayed for luciferase activity at 24 h post transfection (hpt.). **(C)** Effect of viral proteins on IFN-βpromoter activation. HEK293T cells were co-transfected with the Firefly luciferase reporter plasmids pIFN-β-luc, the Renilla luciferase control plasmid, pRL-TK, and viral protein expressing plasmids. Empty plasmids were used as controls. At 24 hpt., cells were treated with SeV (MOI=1) for 12 h; data were normalized using non-stimulated samples to obtain fold induction. **(D)** ISRE promoter luciferase assay. HEK293T cells were co-transfected with pISRE-luc, the internal control plasmid, pRL-TK, and viral protein expressing plasmids. At 24 hpt., cells were treated with 100 ng/µL IFN-α for 12 h; thereafter, dual-luciferase reporter assays were carried out. Results were shown as Mean ± SD. Statistical significance was determined by comparison to the Flag-N1 using one-way ANOVA with Dunnett’s correction, *p < 0.05, **p < 0.01, ***p < 0.001. The data shown are representative of 3 independent experiments.

To screen the viral proteins of SARS-CoV-2 that regulate type I interferon responses, we cloned 27 SARS-CoV-2 genes (**Figure 1A**) into a mammalian expression vector, Flag-N1. After transfection of these constructs into HEK293T cells, individual viral proteins were expressed with a C-terminal Flag tag. Western blotting revealed that 22 viral proteins, except for nsp3, nsp11, ORF3b, ORF7b, and ORF10, were expressed, although at different levels (**Figure 1A**). To identify the SARS-CoV-2 proteins that inhibit IFN-β production and signaling, the overexpression of each viral protein on ISRE promoter activation was tested in HEK293T cells. As shown in **Figure 1B**, the ISRE promoter was activated by the expression of the N-terminal 2-CARD domain of RIG-I (RIG-IN), and nsp2, nsp4, nsp7, nsp9, nsp15, E, M, N, S, ORF7a, ORF8, 3CLpro, and Helicase proteins significantly inhibited the activity of the ISRE promoter. The inhibitory effect on ISRE activity may rely on two steps: 1) suppression of IFN production and 2) inhibition of IFN downstream signaling. To further dissect the effect of these viral proteins on IFN-β promoter activation, HEK293T cells were treated with SeV for 12 h and assayed for luciferase signals to quantify the activation of the IFN-β promoter. Eight proteins significantly suppressed IFN-I production (**Figure 1C**). Among them, nsp7, nsp15, M, N, 3CLpro, and Helicase produced obvious inhibition, whereas nsp2 and nsp4 had lesser inhibitory effects (**Figure 1C**).

Next, the effects of viral proteins on IFN-α-induced ISRE promoter activity was measured. We found that nsp4, nsp7, nsp9, E, S, ORF7a, ORF8, and Helicase proteins had significant inhibitory effects on IFN downstream signaling (**Figure 1D**). These results suggest that multiple SARS-CoV-2 proteins antagonize IFN-I production or IFN signaling to contribute to evasion of the host innate immune response.

### Viral Proteins Antagonize IFN-β Production

**Figure 2:**
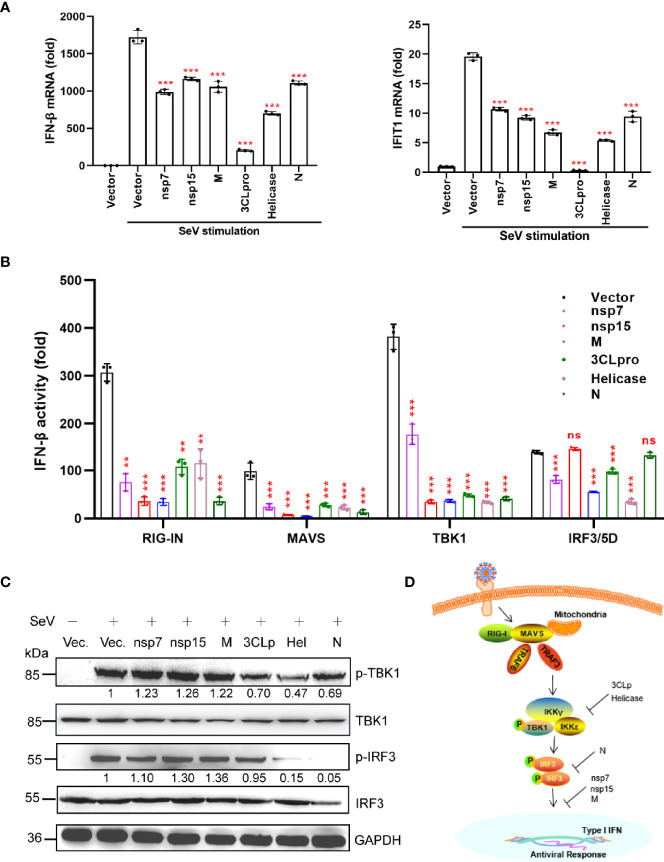
A subset of viral proteins antagonize IFN-β production. **(A)** HEK293T cells cultured in 24-well plates (1 × 10^5^ cells per well) were transfected with Flag-N1 empty vector (200 ng) or the SARS-CoV-2 protein plasmids (200 ng). At 24 h after transfection, cells were stimulated by SeV (MOI=1), and at 12 h after stimulation, the cells were harvested for RNA extraction and subsequent RT-qPCR analysis to assess the expression of IFN-β or ISG (IFIT1). **(B)** The Flag-N1 empty vector and the SARS-CoV-2 protein plasmids (100 ng) were transfected with the indicated combinations of plasmids expressing RIG-IN (10 ng), MAVS (10 ng), TBK1 (100 ng), and IRF3-5D (10 ng) into HEK293T cells cultured in 96-well plates (0.5 × 10^5^ cells per well). The IFN-β-Luc (50 ng) plasmids were co-transfected to assess the activation of IFN promoter and the pRL-TK (5 ng) was transfected as an internal control. Dual luciferase assays were performed 36 hpt. Results were shown as Mean ± SD. Statistical significance was assessed *via* comparison to the Flag-N1 control using one-way ANOVA with Dunnett’s correction, **p < 0.01, ***p < 0.001. ns, not significant. The data shown are representative of 3 independent experiments. **(C)** Phosphorylation of IRF3 and TBK1. HEK293T cells were transfected with viral protein-encoding plasmids (1 µg), treated with SeV for 12 h, and analyzed for phosphorylated IRF3 (anti-p-IRF3 at S396), total IRF3 (anti-IRF3), phosphorylated TBK1 (anti-p-TBK1 at S172), total TBK1 (anti-TBK1), and GAPDH (anti-GAPDH) by western blotting. Representative blots of three independent experiments are shown. **(D)** Summary of the antagonism of IFN-I production. The potential inhibitory steps are indicated for individual viral proteins.

To further validate the inhibitory effect of nsp7, nsp15, M, 3CLpro, Helicase, and N proteins on IFN production, we measured the SeV-induced mRNA levels of *IFN-β* and *IFIT1* (ISG) in HEK293T cells expressing individual viral proteins by quantitative real-time PCR (qRT-PCR) (**Figure 2A**). Based on our findings, all the selected viral proteins suppressed the mRNA expression of *IFN-β*, as well as its downstream target gene (*IFIT1*).

We then determined whether the SARS-CoV-2 proteins interfere with the activation of the dsRNA-sensing RIG-I pathway induced by the overexpression of the components of the signaling cascade. To determine the antagonizing steps where nsp7, nsp15, M, 3CLpro, Helicase, and N proteins block the RIG-I pathway, we transfected the cells with plasmids encoding key signaling proteins involved in the RIG-I pathway and determined the activation of the IFN-β promoter in the presence of different viral proteins. As shown in **Figure 2B**, the overexpression of all six proteins inhibited RIG-IN, MAVS, and TBK1-triggered IFN promoter activation. Interestingly, nsp7, M, 3CLpro, and Helicase suppressed IRF3/5D (a constitutively active IRF3 mutant)-activated IFN-β promoter activity; however, nsp15 and N protein did not have this effect. These findings demonstrate that the nsp15 and N proteins inhibited IFN-β production upstream of IRF3 activation, while nsp7, M, 3CLpro, and Helicase could inhibit IFN-β production at the level of or downstream of IRF3 activation.

Phosphorylation of IRF3 and TBK1 is the hallmark of their activation, which is essential for type I IFN induction during viral infection. Therefore, we investigated the effect of the above six SARS-CoV-2 proteins on IRF3 and TBK1 phosphorylation. We found that only 3CLpro, Helicase, and N proteins significantly inhibited SeV-induced IRF3 or TBK1 phosphorylation (**Figure 2C**). These results indicated that nsp7, nsp15, and M proteins may antagonize IFN-β production by inhibiting the nuclear translocation of IRF3, instead of suppressing its phosphorylation (**Figure 2D**). These results also suggested that 3CLpro and Helicase proteins antagonized IFN-β production by inhibiting the phosphorylation of TBK1 (**Figure 2D**), while N protein mainly inhibited the phosphorylation of IRF3 (**Figure 2D**).

### M Protein Inhibits the Interaction Between KPNA6 and IRF3

**Figure 3:**
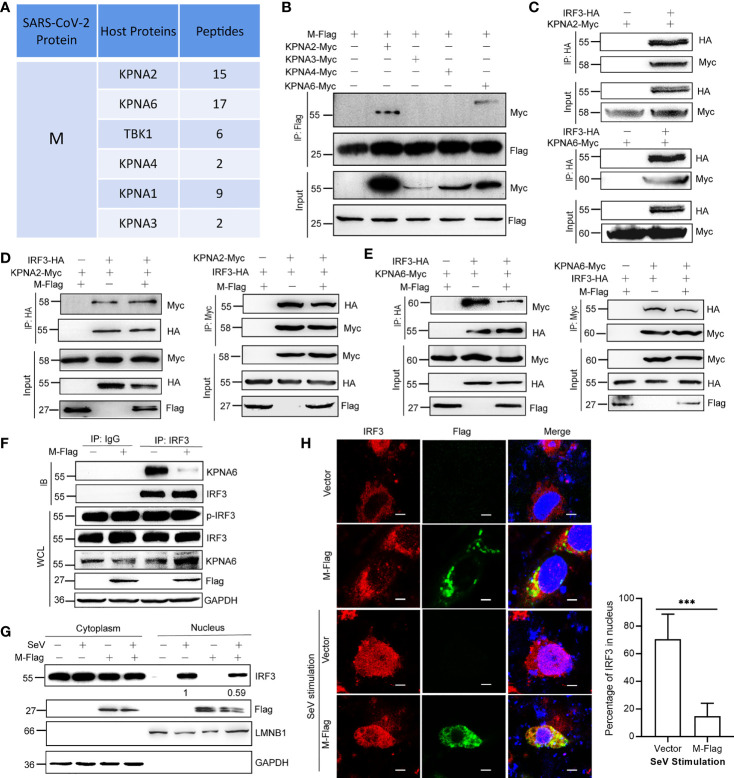
M protein inhibits the interaction between KPNA6 and IRF3. **(A)** Partial results of the IP-MS assay using Flag-tagged M protein as a bait. HEK293T cells were co-transfected with plasmid encoding Flag-tagged SARS-CoV-2 M protein. At 24 hpt., cells were treated with SeV for 12 h Co-IP was performed by incubating the cell lysates with anti-Flag magnetic beads overnight, and eluted proteins were subjected to western blotting verification to confirm successful IP of viral proteins. The elution mixture was processed for protein identification by Mass Spectrometry. The results were analyzed by Proteome Discoverer 2.2 software. **(B)** Co-IP of M and IPed KPNA proteins. HEK293T cells were co-transfected with Flag-tagged SARS-CoV-2 M plasmid and each Myc-tagged KPNA2/KPNA3/KPNA4/KPNA6 plasmid. At 36 hpt., Co-IP was performed by incubating the lysates with the anti-Flag antibody magnetic beads overnight. After extensive washing, the eluate was analyzed by western blotting with indicated antibodies. **(C)** Co-IP of IRF3 and KPNA2 or KPNA6. HEK293T cells were co-transfected with HA-tagged IRF3 plasmids and Myc-tagged KPNA2 or KPNA6 plasmids or empty plasmids. At 36 hpt., Co-IP was performed by incubating the lysates with the anti-HA antibody overnight before the addition of magnetic beads. **(D)** HEK293T cells were transfected with plasmids encoding IRF3-HA and KPNA2-Myc, together with or without M-Flag. The cell lysate was subjected to a Co-IP assay using anti-HA or anti-Myc. **(E)** HEK293T cells were transfected with plasmids encoding IRF3-HA and KPNA6-Myc, together with or without M-Flag. Then the cell lysate was subjected to a Co-IP assay using anti-HA or anti-Myc. **(F)** Interaction between endogenous IRF3 and KPNA6 in the presence of M protein. HEK293T cells were transfected with M-Flag for 24 h and then treated with SeV for another 12 h, the cells were harvested and subjected to a Co-IP assay using IgG or IRF3 antibody. **(G)** HEK293T cells were transfected with M-Flag for 24 h and treated with SeV for 12 h. IRF3 in the nuclear fractions or the cytoplasmic was determined by immunoblotting analyses. GAPDH and Lamin B1 served as cytoplasmic and nuclear protein controls, respectively. **(H)** Nuclear translocation of IRF3. HeLa cells were transfected with M-Flag or empty vector. At 24 hpt., cells were fixed with 4% paraformaldehyde and permeabilized with 0.1% Triton X-100. After blocking with PBS containing 2% fetal bovine serum (FBS), the cells were probed with primary antibodies (anti-Flag and anti-IRF3) and secondary antibodies (anti-Alexa Fluor 488 and anti-Alexa Fluor 648). Scale bar, 10 µm. Representative blots and fluorescence pictures of 3 independent experiments were shown. The percentages of IRF3 in the nucleus (out of total IRF3 signal) were quantified by ImageJ software. ***P<0.001, *t*-test.

To further explore the underlying mechanisms by which viral proteins interfere with the IFN pathway, we performed a protein immunoprecipitation followed by mass spectrometry (IP-MS) experiments to identify the host proteins that interact with the viral protein (Additional File 2). From the mass spectrum results, we found that M protein interacted with many host nuclear transport factors, such as KPNA2, KPNA3, KPNA4, and KPNA6 (**Figure 3A**). Karyopherin α 1-6 (KPNA1-6) are key factors for the nuclear translocation of activated IRF3, IRF7, and STAT1, and the nuclear translocation of phosphorylated IRF3 is a pivotal step in the activation of IFN transcription. Considering that M does not decrease the p-IRF3 level (**Figure 2C**), we speculated that M protein might involve in the function of importins. To verify these interactions, we performed a protein co-immunoprecipitation (Co-IP) assay, and the results showed that the M protein directly interacts with KPNA2 and KPNA6 (**Figures 3B, C**). Importantly, the Co-IP assay revealed that the M protein interfered with the binding of KPNA6 to IRF3, but not the binding of KPNA2 to IRF3 (**Figures 3D, E**). We also confirmed that M protein significantly inhibited the binding of endogenous IRF3 to KPNA6 (**Figure 3F**). Thereafter, we determined the effect of the SARS-CoV-2 M protein on SeV-induced IRF3 nuclear translocation. As shown in **Figure 3G**, SeV-induced IRF3 nuclear translocation was disturbed by the overexpression of the M protein in HEK293T cells in a western blotting assay. This result was confirmed using fluorescent confocal microscopy. In non-stimulated HEK293T cells, both IRF3 and M were primarily localized to the cytoplasm (**Figure 3H**). Once cells were stimulated with SeV, IRF3 was translocated from the cytoplasm to the nucleus; however, most of the IRF3 remained in the cytoplasm in the presence of the M protein (**Figure 3H**). Taken together, these results suggest that the M protein inhibits IFN-β production by binding to KPNA6 and blocking IRF3 nuclear translocation mediated by KPNA6.

### A Subset of Viral Proteins Antagonize the JAK-STAT Signaling Pathway

**Figure 4:**
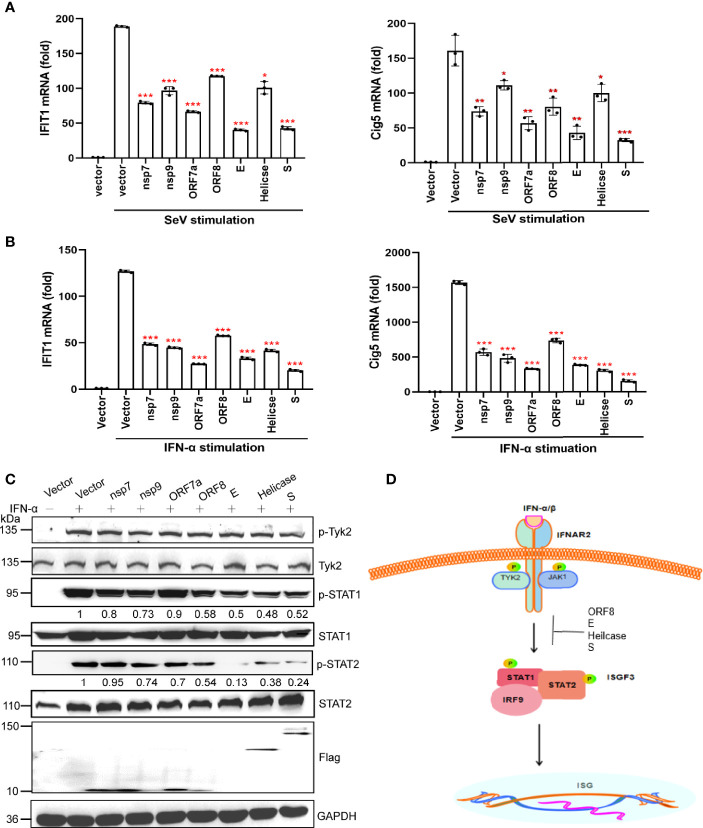
A subset of viral proteins antagonize the JAK-STAT signaling pathway. **(A, B)** HEK293T cells cultured in 24-well plates (1 × 10^5^ cells per well) were transfected with Flag-N1 empty vector (200 ng) or individual SARS-CoV-2 protein encoding plasmids (200 ng). At 24 hpt., cells were stimulated by SeV infection **(A)** or IFN-α stimulation **(B)** as indicated. At 12 h after stimulation, the cells were harvested for RNA extraction and subsequent RT-qPCR analysis to assess the activation of ISGs (IFIT1 and Cig5). Results were shown as Mean ± SD. Statistical significance was assessed *via* comparison to the Flag-N1 control using one-way ANOVA with Dunnett’s correction, *p < 0.05, **p < 0.01, ***p < 0.001. The data shown are representative of 3 independent experiments. **(C)** Inhibition of STAT1, STAT2, and TYK2 phosphorylation. HEK293T cells were transfected with viral protein expressing plasmids. At 24 hpt., cells were treated with 100 ng/uL IFN-α for 12 h and analyzed by western blotting using anti-phosphorylated STAT1 at Y701, anti-total STAT1, anti-phosphorylated STAT2 at Y690, anti-total STAT2, anti-phosphorylated TYK2 at Tyr1054, and anti-total TYK2. Protein band intensity was quantified using ImageJ software. Representative blots of three independent experiments are shown. **(D)** Summary of the antagonism of IFN-I downstream signaling. Inhibitory steps are indicated for individual viral proteins.

We measured the effects of nsp7, nsp9, ORF7a, ORF8, E, Helicase, and S proteins on the IFN downstream JAK-STAT signaling pathway. We first examined the effect of these seven viral proteins on downstream ISGs (*IFIT1* and *Cig5)* expression by qRT-PCR. Based on the results, these viral proteins significantly suppressed the expression of *IFIT1* and *Cig5* induced by SeV or IFN-α (**Figures 4A, B**).

We performed western blotting to determine the effect of the SARS-CoV-2 protein on the activation of the JAK-STAT signaling pathway. Nsp7, nsp9, and ORF8 were found to slightly inhibit STAT1 phosphorylation, whereas E, Helicase, and S proteins significantly suppressed the phosphorylation of STAT1 and STAT2 (**Figure 4C**). These results suggest that SARS-CoV-2 may inhibit IFN-I downstream signaling by suppressing STAT1 phosphorylation by nsp7, nsp9, ORF8, E, Helicase, and S, and inhibiting STAT2 phosphorylation by E, Helicase, and S (**Figure 4D**).

### S Protein Antagonizes the Activation of STAT1

**Figure 5:**
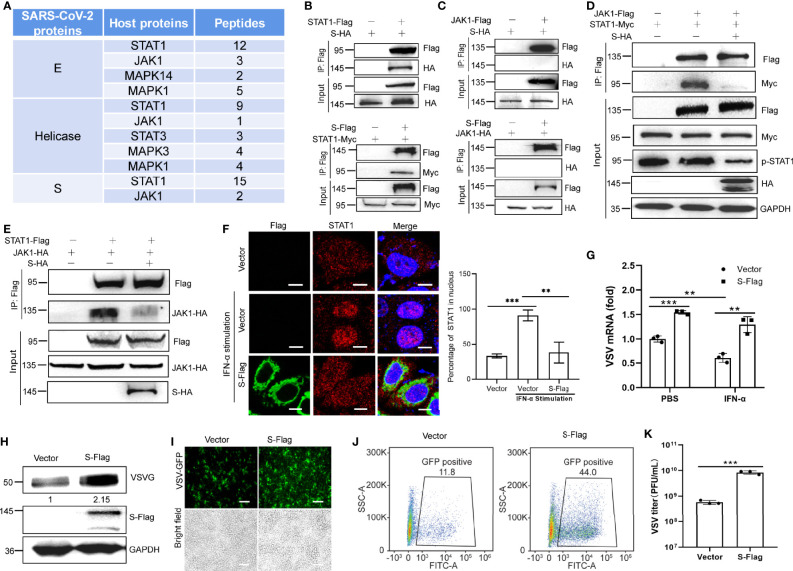
S protein antagonizes the activation of STAT1. **(A)** Partial results of the IP-MS assay with the SARA-CoV-2 proteins. HEK293T cells were co-transfected with Flag-tagged SARS-CoV-2 protein encoding plasmids. At 24 hpt., cells were treated 100 ng/µL IFN-α for 12 h Co-IP was performed by incubating cells with the anti-Flag magnetic beads, and eluted proteins were subjected to western blotting verification to confirm successful IP of viral proteins. The elution mixture was processed for protein identification by mass spectrometry. The results were analyzed using Proteome Discoverer 2.2 software. **(B)** Co-IP of S and STAT1. HEK293T cells were co-transfected with HA-tagged S plasmid and Flag-tagged STAT1 plasmid. Co-IP was performed with anti-Flag antibody. The reciprocal Co-IP was also performed. **(C)** Co-IP of S and JAK1. HEK293T cells were co-transfected with HA-tagged S plasmid and Flag-tagged JAK1 plasmid. Co-IP was performed with anti-Flag antibody. The reciprocal Co-IP was also performed. **(D)** HEK293T cells were transfected with plasmids encoding Flag-tagged JAK1 and Myc-tagged STAT1 together with or without S-HA plasmid. Co-IP assay was performed using anti-Flag. **(E)** The reciprocal Co-IP to test the function of S protein on the interaction between JAK1 and STAT1. **(F)** S protein alters the nuclear translocation of STAT1. Fluorescence micrographs were analyzed using ImageJ. Scale bar, 10 µm. **(G-K)** SARS-CoV-2 S protein facilitates viral replication. HEK293T cells were transfected with the indicated plasmids. At 24 hpt., cells were treated with IFN-α (100 ng/µL) for 12 h RT-qPCR was performed to measure the mRNA replication levels of VSV **(G)**. The replication of VSV was determined by western blot using VSV G antibody **(H)**. GFP-positive cells were visualized by confocal microscopy **(I)** and analyzed by flow cytometry **(J)**. The culture supernatant (6 hpi.) was collected for TCID_50_ assays to measure the titers of extracellular VSV-GFP (PFU/mL) **(K)**. Representative blots **(B-E, H)**, representative confocal imaging **(F, I)** and flow cytometry results **(J)** of three independent experiments are shown. Scale bar, 50 µm. Data from three independent biological replicates were analyzed **(F, G, K)**; Results were shown as Mean ± SD. **p < 0.01, ***p < 0.001, *t*-test.

To determine how E, S, and Helicase inhibit the activities of STAT1 and STAT2, we carried out IP-MS assays to identify signal molecules related to the JAK-STAT signaling pathway that may interact with these viral proteins. Under IFN-α stimulation, JAK1 and STAT1 were pulled out by each of the selected viral proteins (**Figure 5A**). Subsequently, we co-transfected the cells with STAT1-Flag or JAK1-Flag together with the S-HA constructs. Co-IP assay showed that the S protein directly interacted with STAT1 (**Figure 5B**), rather than JAK1 (**Figure 5C**).

JAK1 binds to STAT1 and phosphorylate it; thus, we investigated whether the S protein interferes with the interaction between JAK1 and STAT1. Co-IP assays indicated that the S protein inhibited the binding of JAK1 to STAT1 and subsequently suppressed its phosphorylation (**Figures 5D, E**). Immuno-fluorescence confirmed that S protein inhibited the translocation of STAT1 into the nucleus in HeLa cells under IFN-α stimulation (**Figure 5F**). These results suggest that S inhibits JAK-STAT signaling by suppressing STAT1 phosphorylation.

We then confirmed the suppressive function of the S protein on IFN signaling using a viral replication assay. VSV is commonly used as a model virus to study the effect of IFNs on viral replication. HEK293T cells transfected with an empty vector or the S protein-expressing plasmid were infected with VSV-GFP. The RT-qPCR results indicated that S protein subverted the inhibitory effect of IFN-α on VSV RNA replication (**Figure 5G**). Our western blotting results also showed that GFP expression in HEK293T cells expressing S protein was higher than that in control cells (**Figure 5H**). Confocal microscopy and flow cytometry confirmed that greater numbers of VSV-GFP-positive cells were observed in S protein-expressing cells than in control cells transfected with the empty vector (**Figures 5I, J**). In the culture supernatant of HEK293T cells expressing the S protein, the VSV-GFP titer was markedly higher than that in the supernatant of HEK293T cells transfected with the empty vector (**Figure 5K**). Thus, these results indicate that the S protein suppresses the antiviral efficacy of IFN-α and facilitates the replication of VSV-GFP.

### S Protein Antagonizes JAK-STAT Signaling *via* Its S1 Subunit

**Figure 6:**
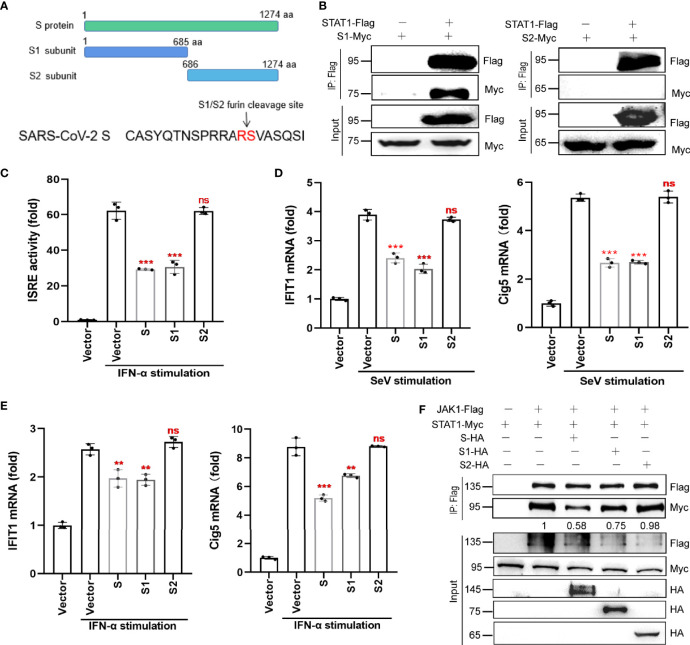
S protein antagonizes host innate antiviral responses *via* its S1 subunit. **(A)** The subunits, S1 and S2, of SARS-CoV-2 S protein. **(B)** Co-IP of STAT1 with S1 or S2 subunit. HEK293T cells were co-transfected with the Flag-tagged STAT1 plasmid and Myc-tagged S1 or S2 plasmid. At 36 hpt., Co-IP was performed by incubating cells with the anti-Flag magnetic beads. After extensive washing, the eluate was analyzed by western blotting with the indicated antibodies. **(C)** The effect of S, S1 and S2 on ISRE promoter activation. HEK293T cells were co-transfected with pISRE-luc, control plasmids, pRL-TK, and S, S1, S2 subunit expressing plasmids. At 24 hpt., cells were treated with 100 ng/µL IFN-α for 12 h; thereafter, dual-luciferase reporter assays were performed. **(D, E)** RT-qPCR analysis was performed to assess the regulation of ISGs (IFIT1 and Cig5) by S protein (or its two subunits) in the presence of SeV infection **(D)** or IFN-α stimulation **(E)**. Results were shown as Mean ± SD. Statistical values were determined *via* comparison to the Flag-N1 control using one-way ANOVA with Dunnett’s correction, **p < 0.01, ***p < 0.001. ns, not significant. **(F)** HEK293T cells were transfected with plasmids encoding JAK1-Flag and STAT1-Myc together with either the plasmids encoding S-HA, S1-HA, S2-HA, or empty vector. At 48 hpt., the cells were harvested and subjected to a Co-IP assay using anti-Flag antibody. The immunoprecipitated proteins and the inputs were separated by SDS-PAGE and probed with anti-HA, anti-Flag, and anti-Myc. The data shown are representative of 3 independent experiments.

The S protein has two subunits, S1 and S2. The S1 subunit, which contains the receptor binding domain (RBD), is responsible for binding to the host cell receptor ACE2, while the S2 subunit is responsible for the fusion of the virus with the cell membrane. To determine which subunit of the S protein can antagonize IFN-I signaling, we generated truncated variants of SARS-CoV-2 S, S1, and S2 (**Figure 6A**). Co-IP revealed that S1, but not S2, interacted with STAT1 (**Figure 6B**). Luciferase reporter assay confirmed that the S1 subunit and the full-length S protein inhibited ISRE promoter activation, whereas the S2 subunit did not (**Figure 6C**).

We also analyzed the effect of the S1 and S2 subunits on the mRNA expression of selected ISGs (*IFIT1* and *Cig5*) by qRT-PCR. Only the S1 subunit and the full-length S protein inhibited SeV- or IFN-α-induced *IFIT1* and *cig5* expression (**Figures 6D, E**). Finally, western blotting results showed that, similar to the full-length S protein, the S1 subunit disrupted the interaction between JAK1 and STAT1 (**Figure 6F**), but the S2 subunit did not. These results suggest that S protein’s antagonism of host innate immune responses mainly dependent on its S1 subunit.

## Discussion

Clinical studies have reported a lack of IFN response despite robust cytokine and chemokine production in COVID-19 patients, consistent with the *in vitro* observation that SARS-CoV-2 infection does not induce significant IFN production (Arunachalam et al., 2020; Blanco-Melo et al., 2020). Further evidence suggests that IFN production is delayed in patients but is not absent. In early adult clinical studies, the use of human IFN (rIFN-α, nasal spray, or oral lozenges) has been demonstrated to relieve acute respiratory illness (Gao et al., 2010; Bennett et al., 2013). IFN-I is used in combination with other drugs for the treatment of COVID-19. The results obtained to date suggest that this agent may be effective and safe for COVID-19 prophylaxis (Nitulescu et al., 2020). Our results indicate that different viral proteins antagonize different steps of the IFN-I pathway, partially explaining the attenuation of the IFN-I response in patients with COVID-19.

We found that nsp7 and nsp15 had obvious inhibitory effects on the production of IFN-I, but not on the activation of TBK1 and IRF3. Gordon et al. also showed that nsp15 suppresses IFN-I production through the E3 ligase, RNF41 (Gordon et al., 2020). Nsp7 inhibits both IFN-I production and IFN signaling. These phenotypes are consistent with those in our current study; however, the specific mechanisms underlying them need to be further investigated (Yuen et al., 2020).

Nsp13, also known as Helicase protein, is a highly conserved member of the RNA Helicase superfamily. Based on our findings, the SARS-CoV-2 Helicase protein inhibits the production of IFN-I by affecting the activation of TBK1, and the N protein affects the production of IFN-I by antagonizing the activation of IRF3. Consistent with our results, Xia et al. reported that nsp13 associates with TBK1, leading to reduced TBK1 phosphorylation and IRF3 inactivation (Xia et al., 2020); SARS-CoV-2 N protein has been found to antagonize IFN-I production by interacting with RIG-I (Chen et al., 2020).

Previous studies have shown that the coronavirus M protein can directly interact with RIG-I, TBK1, IKKϵ, and TRAF3 to inhibit IRF3 activation (Siu et al., 2009; Fu et al., 2021). Our results indicate that the M protein does not significantly inhibit the phosphorylation of TBK1 or IRF3; instead, it impeded the nuclear translocation of IRF3 through the nuclear transporter, KPNA6, and attenuated IFN production. In our Nuclear/Cytoplasmic fractionation assay, we found that M protein was also detected in the nucleus. Bioinformatic analysis indicated that M protein is associated with membranes and could locate in cellular membrane associated structures such as endoplasmic reticulum (ER) membrane. Thus, we hypothesized that a portion of the M protein may bind to the surface of the nuclear membrane and interrupt the nuclear interaction of KPNA6 and IRF3. Shi *et al*. also found that the SARS-CoV-2 M protein significantly blocked the phosphorylation of STAT1, leading to a decrease in ISG expression (Xia et al., 2020), however, this phenomenon was not observed in our study.

We also identified SARS-CoV-2 proteins that antagonize IFN downstream signaling. Although nsp7 and ORF7a suppress the activation of the ISRE promoter, they do not directly influence the activation of STAT1 and STAT2. Nsp9 and ORF8 slightly inhibited the activation of STAT1, and Helicase, E, and S proteins remarkably inhibited the activation of STAT1 and STAT2. Importantly, the S protein affects the activation of STAT1 by inhibiting the interaction between JAK1 and STAT1. A study by Shi et al. also suggested that both ORF7a and ORF7b strongly blocked IFN-I downstream signaling pathways. In their experimental settings, ORF7a only inhibited STAT2 phosphorylation, whereas ORF7b inhibited both STAT1 and STAT2 phosphorylation (Xia et al., 2020). There are multiple studies showing that ORF8 blocks IFN-β production and signaling pathways in a dose-dependent manner, which is consistent with our findings; however, the underlying mechanism remains to be determined (Li et al., 2020a; Li et al., 2020b). Additional studies have shown that ORF8 promotes viral growth, partially by preventing IFN-β and RIG-I pathway-mediated ISREs, ISGs, and NF-κB transcription (Li et al., 2020b). In another study by Lei et al., when cells were stimulated by SeV, MDA5, or RIG-I, SARS-CoV-2 M protein significantly inhibited IFN-I production; N protein inhibited the IFN-I signaling pathway by reducing the phosphorylation of STAT1 and STAT2; S protein inhibited the IFN-I signaling pathway. However, most of the mechanisms underlying these inhibitions remain unclear (Lei et al., 2020; Mu et al., 2020). Our data revealed that S protein suppresses STAT1 phosphorylation by directly binding to STAT1 vis its S1 subunit. Mutations in the S protein, especially in the S1 subunit, are considered the most common reason for the immune evasion of SARS-CoV-2. We hypothesize that mutations in the S gene not only influence the neutralization ability of antibodies, but also affect their ability to antagonize IFN signaling. Further studies are needed to address the suppressive effects of the S protein on IFN-I signaling in newly emerging SARS-CoV-2 variants.

Although many of our data are consistent with published results, some novel findings regarding the evasion mechanisms of SARS-CoV-2 on IFN signaling were also observed. The differences between our results and those of others may be due to the use of different experimental systems, such as the cell lines used, the amount of plasmid transfected per well, and the transfection time (Xia et al., 2020). Another reason may be that the prokaryotic plasmids that we used as templates for amplifying all viral ORFs had undergone different optimizations that may have impacted our experimental results. In addition, different stimulus conditions may produce inconsistent results. Thus, multiple validations using different experimental methods are required. Ideally, performing these experiments with SARS-CoV-2 infection would be more accurate.

In summary, we identified SARS-CoV-2 proteins that antagonize IFN-I production and the IFN-I signaling pathway. The antagonistic steps of each identified protein were mapped onto different components of the IFN-I signaling cascade. Importantly, our study reveals for the first time that SARS-CoV-2 M protein inhibits IRF3 nuclear translocation by inhibiting the interaction between KPNA6 and IRF3, and S protein inhibits STAT1 activation and nuclear translocation by inhibiting the interaction of JAK1 and STAT1. This finding may provide promising targets for antiviral intervention. Our findings provide new evidence for the interaction between SARS-CoV-2 and mammalian host cells and contribute to a better understanding of the pathogenesis of COVID-19.

## Data Availability Statement

The original contributions presented in the study are included in the article/**Supplementary Material**. Further inquiries can be directed to the corresponding authors.

## Author Contributions

JD and KW conceived of and designed the project. QZ, ZC, and CH performed most of the experiments and data analyses. JS, MX, TF, and WP helped with data analysis and discussions. JD and QZ wrote the manuscript. All authors contributed to the article and approved the submitted version.

## Funding

This work was supported by the Priority Academic Program Development of Jiangsu Higher Education Institutions, National Natural Science Foundation of China (31770933, 81971917, 32170142), Jiangsu Natural Science Foundation (BK20211310), Open Project Fund from State Key Laboratory of Genetic Engineering, Fudan University (SKLGE1903), and a grant from Soochow Securities. The funders had no role in the study design, data collection and analysis, decision to publish, or preparation of the manuscript.

## Conflict of Interest

The authors declare that the research was conducted in the absence of any commercial or financial relationships that could be construed as a potential conflict of interest.

## Publisher’s Note

All claims expressed in this article are solely those of the authors and do not necessarily represent those of their affiliated organizations, or those of the publisher, the editors and the reviewers. Any product that may be evaluated in this article, or claim that may be made by its manufacturer, is not guaranteed or endorsed by the publisher.
