# Supplementary material for: Severe Acute Respiratory Syndrome Coronavirus 2 (SARS-CoV-2) Membrane (M) and Spike (S) Proteins Antagonize Host Type I Interferon Response
Source: Front Cell Infect Microbiol. 2021 Dec 7;11:766922. doi: 10.3389/fcimb.2021.766922 (PMC8688923; doi:10.3389/fcimb.2021.766922)
Supplement: Supplementary file 1 [file Table_1.docx]

**Table S1. Sequences of Oligo-primers used in this study.**

| **Number** | **Sequence (5’-3’)** | **Purpose** |
| --- | --- | --- |
| P1 | GCTTCGAATTCTGCAGTCGACACCATGGAGAGCCTGGTTCC | Forward primer for constructing plasmid Flag-N1-nsp1 |
| P2 | ATGGTGGCGACCGGTGGATCCCGGCCACCATTCAGTTCAC | Reverse primer for constructing plasmid Flag-N1-nsp1 |
| P3 | GCTTCGAATTCTGCAGTCGACACCATGGCGTACACCCGTTATGTG | Forward primer for constructing plasmid Flag-N1-nsp2 |
| P4 | ATGGTGGCGACCGGTGGATCCCGGCCACCTTTCAGGGTGAAG | Reverse primer for constructing plasmid Flag-N1-nsp2 |
| P5 | GCTTCGAATTCTGCAGTCGACACCATGAAGATCGTGAACAACTGG | Forward primer for constructing plasmid Flag-N1-nsp4 |
| P6 | ATGGTGGCGACCGGTGGATCCCGCTGCAGAACCGCGCTGG | Reverse primer for constructing plasmid Flag-N1-nsp4 |
| P7 | GCTTCGAATTCTGCAGTCGACACCATGAGCGCGGTGAAGCGT | Forward primer for constructing plasmid Flag-N1-nsp6 |
| P8 | ATGGTGGCGACCGGTGGATCCCGTTGAACGGTCGCCACTTTGA | Reverse primer for constructing plasmid Flag-N1-nsp6 |
| P9 | GCTTCGAATTCTGCAGTCGACACCATGAGCAAGATGAGCGACGT | Forward primer for constructing plasmid Flag-N1-nsp7 |
| P10 | ATGGTGGCGACCGGTGGATCCCGCTGCAGGGTCGCACGGTTA | Reverse primer for constructing plasmid Flag-N1-nsp7 |
| P11 | GCTTCGAATTCTGCAGTCGACACCATGGCGATCGCGAGCGA | Forward primer for constructing plasmid Flag-N1-nsp8 |
| P12 | ATGGTGGCGACCGGTGGATCCCGTTGCAGCTTAACCGCGC | Reverse primer for constructing plasmid Flag-N1-nsp8 |
| P13 | GCTTCGAATTCTGCAGTCGACACCATGAACAACGAGCTGAGCCCG | Forward primer for constructing plasmid Flag-N1-nsp9 |
| P14 | ATGGTGGCGACCGGTGGATCCCGCTGCAGACGAACGGTCGCC | Reverse primer for constructing plasmid Flag-N1-nsp9 |
| P15 | GCTTCGAATTCTGCAGTCGACACCATGGCGGGTAACGCGAC | Forward primer for constructing plasmid Flag-N1-nsp10 |
| P16 | ATGGTGGCGACCGGTGGATCCCGTTGCAGCATCGGCTCACG | Reverse primer for constructing plasmid Flag-N1-nsp10 |
| P17 | GCTTCGAATTCTGCAGTCGACACCATGAGCCTGGAAAACGTTG | Forward primer for constructing plasmid Flag-N1-nsp15 |
| P18 | ATGGTGGCGACCGGTGGATCCCGTTGCAGTTTCGGATAGAAGGTT | Reverse primer for constructing plasmid Flag-N1-nsp15 |
| P19 | GCTTCGAATTCTGCAGTCGACACCATGAGCAGCCAGGCGTG | Forward primer for constructing plasmid Flag-N1-nsp16 |
| P20 | ATGGTGGCGACCGGTGGATCCCGGTTGTTAACCAGCACATCGCTG | Reverse primer for constructing plasmid Flag-N1-nsp16 |
| P21 | GCTTCGAATTCTGCAGTCGACACCATGTACAGCTTCGTGAGCGAG | Forward primer for constructing plasmid Flag-N1-E |
| P22 | ATGGTGGCGACCGGTGGATCCCGAACCAGCAGATCCGGCACAC | Reverse primer for constructing plasmid Flag-N1-E |
| P23 | GCTTCGAATTCTGCAGTCGACACCATGGCGGACAGCAACGGTA | Forward primer for constructing plasmid Flag-N1-M |
| P24 | ATGGTGGCGACCGGTGGATCCCGCTGAACCAGCAGCGCAATGTTA | Reverse primer for constructing plasmid Flag-N1-M |
| P25 | GCTTCGAATTCTGCAGTCGACACCATGAGCGACAACGGTCCG | Forward primer for constructing plasmid Flag-N1-N |
| P26 | ATGGTGGCGACCGGTGGATCCCGCGCCTGGGTGCTATCCG | Reverse primer for constructing plasmid Flag-N1-N |
| P27 | GCTTCGAATTCTGCAGTCGACaccATGTTCGTTTTTCTGGTGCTGCTGC | Forward primer for constructing plasmid Flag-N1-S |
| P28 | ATGGTGGCGACCGGTGGATCCCGGGTGTAGTGCAGTTTCACAC | Reverse primer for constructing plasmid Flag-N1-S |
| P29 | GCTTCGAATTCTGCAGTCGACACCATGGACCTGTTCATGCGTAT | Forward primer for constructing plasmid Flag-N1-ORF3a |
| P30 | ATGGTGGCGACCGGTGGATCCCGCAGCGGAACGCTGGTGG | Reverse primer for constructing plasmid Flag-N1-ORF3a |
| P31 | GCTTCGAATTCTGCAGTCGACACCATGTTCCACCTGGTGGATTT | Forward primer for constructing plasmid Flag-N1-ORF6 |
| P32 | ATGGTGGCGACCGGTGGATCCCGATCAATTTCCATCGGTTGTT | Reverse primer for constructing plasmid Flag-N1-ORF6 |
| P33 | GCTTCGAATTCTGCAGTCGACACCATGAAAATCATTCTGTTTCT | Forward primer for constructing plasmid Flag-N1-ORF7a |
| P34 | ATGGTGGCGACCGGTGGATCCCGTTCGGTTTTACGCTTCAGGG | Reverse primer for constructing plasmid Flag-N1-ORF7a |
| P35 | GCTTCGAATTCTGCAGTCGACACCATGAAATTCCTGGTGTTTC | Forward primer for constructing plasmid Flag-N1-ORF8 |
| P36 | ATGGTGGCGACCGGTGGATCCCGGATGAAGTCCAGAACCACAC | Reverse primer for constructing plasmid Flag-N1-ORF8 |
| P37 | GCTTCGAATTCTGCAGTCGACACCATGAGCGGTTTCCGTAAGATG | Forward primer for constructing plasmid Flag-N1-3CLpro |
| P38 | ATGGTGGCGACCGGTGGATCCCGTTGAAAGGTCACACCGCTGC | Reverse primer for constructing plasmid Flag-N1-3CLpro |
| P39 | GCTTCGAATTCTGCAGTCGACACCATGGCGGTGGGTGCGTG | Forward primer for constructing plasmid Flag-N1-Helicase |
| P40 | ATGGTGGCGACCGGTGGATCCCGCTGCAGGGTCGCAACGTTAC | Reverse primer for constructing plasmid Flag-N1-Helicase |
| P41 | GCTTCGAATTCTGCAGTCGACACCATGGCGGAAAACGTGAC | Forward primer for constructing plasmid Flag-N1-Gunaine-N7 |
| P42 | ATGGTGGCGACCGGTGGATCCCGTTGCAGACGGGTGAAGGTGT | Reverse primer for constructing plasmid Flag-N1-Gunaine-N7 |
| P43 | GCTTCGAATTCTGCAGTCGACACCATGAGCGCGGACGCGCA | Forward primer for constructing plasmid Flag-N1-Polymerase |
| P44 | ATGGTGGCGACCGGTGGATCCCGCTGCAGAACGGTGTGCGGGG | Reverse primer for constructing plasmid Flag-N1-Polymerase |
| P45 | GCTTCGAATTCTGCAGTCGACaccATGTTCGTTTTTCTGGTGCTGCTGC | Forward primer for constructing plasmid HA-N1-S |
| P46 | ATGGTGGCGACCGGTGGATCCCGGGTGTAGTGCAGTTTCACAC | Reverse primer for constructing plasmid HA-N1-S |
| P47 | GCTTCGAATTCTGCAGTCGACACCATGTCTCAGTGGTACGAACTTCAG | Forward primer for constructing plasmid Flag-N1-STAT1 |
| P48 | ATGGTGGCGACCGGTGGATCCCGCACTTCAGACACAGAAATCAACTC | Reverse primer for constructing plasmid Flag-N1-STAT1 |
| P49 | GCTTCGAATTCTGCAGTCGACACCATGTCTCAGTGGTACGAACTTCAG | Forward primer for constructing plasmid Myc-N1-STAT1 |
| P50 | ATGGTGGCGACCGGTGGATCCCGCACTTCAGACACAGAAATCAACTC | Reverse primer for constructing plasmid Myc-N1-STAT1 |
| P51 | GCTTCGAATTCTGCAGTCGACACCATGTTCGTTTTTCTGGTGCTGCTGC | Forward primer for constructing plasmid Myc-N1-S1 |
| P52 | ATGGTGGCGACCGGTGGATCCCGACGCGCACGACGCGGGCT | Reverse primer for constructing plasmid Myc-N1-S1 |
| P53 | GCTTCGAATTCTGCAGTCGACACCATGTTCGTTTTTCTGGTGCTGCTGC | Forward primer for constructing plasmid HA-N1-S1 |
| P54 | ATGGTGGCGACCGGTGGATCCCGACGCGCACGACGCGGGCT | Reverse primer for constructing plasmid HA-N1-S1 |
| P55 | GCTTCGAATTCTGCAGTCGACACCATGAGCGTTGCGAGCCAG | Forward primer for constructing plasmid Myc-N1-S2 |
| P56 | ATGGTGGCGACCGGTGGATCCCGGGTGTAGTGCAGTTTCACAC | Reverse primer for constructing plasmid Myc-N1-S2 |
| P57 | GCTTCGAATTCTGCAGTCGACACCATGAGCGTTGCGAGCCAG | Forward primer for constructing plasmid HA-N1-S2 |
| P58 | ATGGTGGCGACCGGTGGATCCCGGGTGTAGTGCAGTTTCACAC | Reverse primer for constructing plasmid HA-N1-S2 |
| P59 | GCTTCGAATTCTGCAGTCGACACCATAGGGGCGGGAACAGC | Forward primer for constructing plasmid HA-N1-IRF3 |
| P60 | ATGGTGGCGACCGGTGGATCCCGTCATAGCAGGAACCAGT | Reverse primer for constructing plasmid HA-N1-IRF3 |
| P61 | GCTTCGAATTCTGCAGTCGACACCGCGTCGCTGAGCGCAGG | Forward primer for constructing plasmid Flag-N1-JAK1 |
| P62 | ATGGTGGCGACCGGTGGATCCCGTAAAGTCTTTAGTATAT | Reverse primer for constructing plasmid Flag-N1-JAK1 |
| P63 | GCTTCGAATTCTGCAGTCGACACCGCACTGCGAACGCCGGCTG | Forward primer for constructing plasmid Myc-N1-KPNA1 |
| P64 | ATGGTGGCGACCGGTGGATCCCGTTTTCTTTTATCAAGCACCCT | Reverse primer for constructing plasmid Myc-N1-KPNA1 |
| P65 | GCTTCGAATTCTGCAGTCGACACCGTTGACTAGGCCTCGGGG | Forward primer for constructing plasmid Myc-N1-KPNA2 |
| P66 | ATGGTGGCGACCGGTGGATCCCGTGAAGTCAAGAAAAGGGTGGA | Reverse primer for constructing plasmid Myc-N1-KPNA2 |
| P67 | GCTTCGAATTCTGCAGTCGACACCAGTCGGCCCGCGCCTCCCCC | Forward primer for constructing plasmid Myc-N1-KPNA3 |
| P68 | ATGGTGGCGACCGGTGGATCCCGTTTTATCATATTTGATA | Reverse primer for constructing plasmid Myc-N1-KPNA3 |
| P69 | GCTTCGAATTCTGCAGTCGACACCAGATCGAGGCTGCCTCC | Forward primer for constructing plasmid Myc-N1-KPNA4 |
| P70 | ATGGTGGCGACCGGTGGATCCCGTCATTATCACAAGCATTTATT | Reverse primer for constructing plasmid Myc-N1-KPNA4 |
| P71 | GCTTCGAATTCTGCAGTCGACACCATCTTGGATTGCGAACTGGGTC | Forward primer for constructing plasmid Myc-N1-KPNA5 |
| P72 | ATGGTGGCGACCGGTGGATCCCGTACACAAAGTAGATTCTT | Reverse primer for constructing plasmid Myc-N1-KPNA5 |
| P73 | GCTTCGAATTCTGCAGTCGACACCATATTGTCTACTGAAAGC | Forward primer for constructing plasmid Myc-N1-KPNA6 |
| P74 | ATGGTGGCGACCGGTGGATCCCGAACTTTGCAACAGGTTTTTATT | Reverse primer for constructing plasmid Myc-N1-KPNA6 |
| P75 | CCAACAAGTGTCTCCTCCAAAT | Forward primer for qRT-PCR of human *IFNβ* |
| P76 | AATCTCCTCAGGGATGTCAAAGT | Reverse primer for qRT-PCR of human *IFNβ* |
| P77 | TGACTCTTTTGCCTCTTTCTTCTAA | Forward primer for qRT-PCR of human *IFIT1* |
| P78 | TTCTTGGGGT GCTCTGTGG | Reverse primer for qRT-PCR of human *IFIT1* |
| P79 | TGGGTGCTTACACCTGCTG | Forward primer for qRT-PCR of human *Cig5* |
| P80 | GAAGTGATAGTTGACGCTGGTT | Reverse primer for qRT-PCR of human *Cig5* |
